# Inscribed and expendable: oncological trauma, body memory, and the politics of grievability in Anne Boyer’s *The Undying*

**DOI:** 10.3389/fsoc.2026.1916810

**Published:** 2026-10-13

**Authors:** Immaculate Catherine Anitha Christy, R. K. Jaishree Karthiga

**Affiliations:** School of Social Sciences and Languages, Vellore Institute of Technology, Chennai, India

**Keywords:** feminist illness narrative, grievability, oncological violence, somatic trauma, precarity, traumatic body memory, medical humanities

## Abstract

The study examines the thematic resonances and disjunctions between Medical Humanities, Trauma Studies, and Feminist Critical Theory through a close reading of Anne Boyer’s Pulitzer Prize-winning pathography The Undying. The research argues that the bodies of female cancer patients experience, remember, and resist oncological violence in contrast to late-capitalist biomedical discourse. Drawing on Bessel van der Kolk’s clinical account of implicit, non-declarative body memory, understood here not as a diagnostic claim about Boyer’s neurobiology but as a way of reading literary form, the study reads Boyer’s syntactically fragmented, recursive prose as both a figuration of chemotherapy-induced somatic violation and a formal strategy to represent what testimony cannot. Judith Butler’s analytics of precarity and grievability are essential to this inquiry, illuminating the role of normative biopolitical regimes in allocating institutional protection, therapeutic resources, and public mourning along intersectional axes of gender, ethnicity, and class, rendering certain oncological bodies structurally non-grievable. This reading of the biopolitical investments conditioned by cancer and by its differential histories is further underscored by the World Health Organization’s Global Breast Cancer Initiative and the United Nations Sustainable Development Goal 3 imperative that oncological treatment be gender equitable and that by 2030 deaths from non-communicable diseases be substantially reduced. Within this context, Boyer’s feminist micro-narratives of somatic trauma, chemotherapeutic toxicity, and institutional abandonment stand in transnational epidemiological and humanistic urgency.

## 1 Introduction

Illness has long been an ambivalent space in literature, particularly in terminal cancer illness such as breast cancer, providing a textual language that does not assume a linear trajectory of survivorship, femininity, or biomedical authority (Sontag, 1978; Frank, 1995; Jutel and Russell, 2023; Fernández Morales, 2020a,b). However, breast cancer in literature through contemporary illness narratives is often limited to a lexicon of heroism, feminine stoicism, and redemptive triumph, a dominant discourse of survivorship that privileges optimism, gratitude, and narratable recovery over an active engagement with the bodily, social, and systemic consequences of cancer’s devastation. In *The Undying: Pain, vulnerability, mortality, medicine, art, time, dreams, data, exhaustion, cancer, and care,* Boyer (2019) made a radical intervention into and against the illness narrative and a concerted critique of the political, economic, and epistemological frameworks through which cancer is culturally narrated and understood across the United States.

Working class and uninsured, Boyer refuses the story of the “brave fighter,” the “grateful survivor,” and the “transformed self.” She foregrounds the experiences of toxicity, institutional abandonment, and labor precarity. Boyer tries to identify the non-biological reasons that cancer causes suffering. Boyer explicitly states that “I do not want to tell the story of cancer in the way I have been taught to tell it” (Boyer, 2019, p. 98). Not only thematically, but methodologically, *The Undying* resists the narrative coherence, redemptive closure, and testimonial legibility that secure the epistemic conventions through which the properties of illness are rendered intelligible in both literary and biomedical discourses.

The intervention the study contributes is an interpretive extension of the political claims already made by Boyer’s memoir․ It elaborates those claims using the frameworks of trauma neuroscience, Butlerian biopolitics, and transnational health policy, none of which Boyer uses per se but all of which help clarify the stakes of her testimony․ The study intends not to challenge what Boyer says about her illness, but rather use her account as the primary evidentiary and theoretical ground for the reading.

In this sense, the syntactically fragmented and formally discontinuous prose of the memoir enacts, rather than describes, the disrupted temporality, fractured cognition, and embodied disorientation of traumatic illness. This ultimately positions the memoir as a type of somatic testimony or as part of the challenge of transcribing bodily suffering into literary expression in ways that exceed the limits of not only narrative conventions but also the epistemological constraints of biomedical discourse (Carel, 2016; Toombs, 1992; Laricchiuta et al., 2023). Thus, the body becomes not only the object of testimony but also its archive, registering trauma in the affective, neurological, and material registers that language can never fully grasp.

*The Undying* is conceived in the fields of Medical Humanities, Trauma Studies, and Feminist Critical Theory as a feminist counter-archive, a site of somatic testimony, theoretical insurgency, and political indictment. Chemotherapy’s somatic violation of the body is theorized as an institutionalized traumatic inscription of bodily violence․ The fragmentation of Boyer’s text is theorized as a literary figuration of the resistance of trauma to verbal capture․ In the spirit of Butler’s (2004, 2009) theorization of precarity and grievability, the study contends that Boyer’s cancer-marked body is made structurally non-grievable by a late-capitalist biomedical epistemology that grants legibility, institutional support, and political standing only to certain kinds of suffering. Two transnational policy frameworks that locate these literary-humanistic claims in a global context are the World Health Organization Global Breast Cancer Initiative (World Health Organization, 2023) and the United Nations Sustainable Development Goal 3 (United Nations, 2015), both of which identify her story with a global pattern of capitalist oncological injustice and health inequality․ The study concludes by establishing an autotheoretical methodology as a major methodological intervention in the Medical Humanities that productively combines somatic testimony, intersectional critique, and policy consciousness to understand and read alongside contemporary illness narratives (Palla et al., 2024; Wang et al., 2023; Davidson et al., 2023).

Undeniably, however, the implications of this question extend far beyond Boyer’s memoir alone to more broadly include questions about whose suffering gets narrated, whose gets institutionalized, and whose gets politically grieved within contemporary medical regimes generally․ From the start of the memoir, Boyer is diagnosed with the subtype triple-negative breast cancer (TNBC), the subtype whose absence of the standard hormone-receptors and HER2 targets leaves it with fewer treatment options and a comparatively unpredictable course (Bianchini et al., 2016)․ Boyer’s story also has elements of class consciousness and critique of the institutional response.

It enacts not only the subject’s disempowerment in the diagnostic appointment, compared to the socioeconomically privileged person who has private time and familial support to attend, but also the way in which this was overcome: a friend who worked for an hourly wage and could not afford to lose the pay drove her to the appointment “on her lunch break” before returning immediately to her own job (Boyer, 2019, p. 22). *The Undying’s* structural argument is that these conditions of oncological care are not only incidental biographical circumstances but also constitutive political realities that determine whether suffering is survivable, recognizable, and grievable (Butler, 2009; Estupiñán Fdez de Mesa et al., 2023; Gutnik et al., 2022).

Reading *The Undying* as a text that theorizes the cancered body not as a site of triumphant transcendence but as a site of embodied precarity, structural violence, and resistant testimony, this essay stages an intervention in the growing body of work exploring how it is possible to theorize and conceptualize the meaning of illness narratives in ways that extend beyond the models of narrative recovery, individual resilience, and inspirational writing, examining the potential of cancer narrative as a means of contesting contemporary regimes of gender, labor, healthcare, and grievability.

## 2 Literature review

### 2.1 Medical humanities, pathography, and illness narrative

The interdisciplinary field of Medical Humanities has incorporated the presence of illness narrative into both literary studies and the medical field․ Charon (2001) defines Narrative Medicine as medicine practiced with narrative competence, or “the ability to acknowledge, absorb, interpret, and act on the stories and plights of others” (p․ 1897)․ This definition entails the irreducibility of patient testimony to strictly biomedical terms․ Several other studies extended Charon’s theoretical work and pedagogical model (Palla et al., 2024; Wang et al., 2023; Jutel and Russell, 2023) by investigating how narrative medicine pedagogy affects clinical empathy, diagnostic accuracy, and patient-centered care․ Frank’s (1995) typology of illness stories, the restitution narrative, the chaos narrative, and the quest narrative are useful for heuristic reading of Boyer’s text. *The Undying* disrupts both the restitution narrative (where illness is seen as a break in the normal state of health that medicine can repair) and the quest narrative (where suffering brings wisdom). Instead, Boyer’s text occupies a structurally ambivalent position, simultaneously formally proximate to the chaos narrative in its syntactic fragmentation and refusal of resolution while politically and theoretically advanced in ways that Frank’s typology does not fully accommodate (De La Parra Fernández, 2021; Fernández Morales, 2020a,b).

Hawkins’ (1999) definition of pathography as first-person illness writing intended for public publication was complicated by intersectional feminists, who say that illness narratives are situated and their social meanings and political functions differ depending on gender, ethnicity, and class. In Jutel and Russell’s (2023) typology of historical and contemporary forms of pathography, illness writing is not only personal testimony but also a genre of cultural critique, subject to specific epistemological and political functions. Boyer’s pathography-as-autotheory expands this generic threshold into a hybrid auto/biographical and literary theoretical memoir that the field of Medical Humanities is only beginning to accommodate (Palla et al., 2024).

Sontag’s thesis, especially her critiques of the common cultural myths surrounding cancer (punishment, invasion, and metaphor for social pathology), remains foundational for the field, but has, in many respects, been sidestepped by intersections of cultural studies with the growing field of intersectional and feminist analyses of the uneven cultural and material (Ehlers and Krupar, 2020; Estupiñán Fdez de Mesa et al., 2023; Fernández Morales, 2020a,b) effects experienced by people along the axes of gender, ethnicity, and class. Boyer explicitly positions her work against Sontag’s impersonal account, writing “Illness as Metaphor is cancer as nothing personal. Sontag does not write ‘I’ and ‘cancer’ in the same sentence” (Boyer, 2019, p. 2).

Manderson and Stirling’s (2007) feminist reading of the mastectomized body works productively for Boyer’s own text, the absent or scarred breast a locus of negotiations between the medical institution, feminist movement, and individual embodiment. Boyer uses her surgery to perform the feminist phenomenological work of Manderson and Stirling, describing how her mastectomy was “decided without ceremony” and left her, in its aftermath, “pretending that my breasts were never there” (Boyer, 2019, p. 39). This sentence performs what Carel (2008) describes as the phenomenological restructuring of bodily self-understanding that illness initiates, the sudden estrangement from one’s own body that the clinical gaze produces.

Beyond the illness narrative represented in such writing, the structure of oncology itself shapes patient experience before the clinical encounter Boyer describes. These differences in outcomes in breast cancer are not fully explained by the biology of the tumors and are also influenced by social and healthcare system-level factors (Wheeler et al., 2013)․ These disparities are influenced by insurance coverage and healthcare access, income, and whether the patient receives guideline-concordant treatment․ The perspective of this structural account of care quality provides the backdrop to the institutional abandonment that Boyer’s memoir dramatizes at the level of individual testimony.

### 2.2 Trauma studies, somatic memory, and neurobiological approaches

Clinical studies of cancer survivorship have separately described psychosocial and post-traumatic sequelae persisting many years after treatment․ Amir and Ramati (2002) found in a matched control study that a higher proportion of long-term breast cancer patients had full and partial PTSD than controls, with much worse physical and psychological quality of life.

A recent review of the literature found that depressive, anxious, and post-traumatic symptoms remain high for Asian American breast cancer survivors after treatment (Tsai et al., 2020)․ Drawing upon this corpus of clinical literature on persistent post-traumatic symptomatology, a trauma-theoretical reading of literary form is developed below.

Since then, trauma theory, which Judith Herman developed in 1992 on the clinical register and Cathy Caruth on the literary register in 1996, has made great advances․ Herman’s work, in particular, established the symptoms of traumatic experience (as undervalued as the symptoms of intrusion, constriction, and hyperarousal) within a feminist framework of analysis of the social conditions responsible for the production and persistence of traumatic injury. Caruth’s representation of trauma as a crisis of representability suggests that trauma cannot be articulated at the moment of its occurrence but emerges belatedly and disrupts the sequential structure of narrative and temporality.

Van der Kolk’s (2014) *The Body Keeps the Score* pushes this theoretical lineage toward neurobiology, proposing that traumatic memory bypasses declarative, language-accessible declarative memory. Van der Kolk (2014) goes on to describe the neurobiology of this theory in The Body Keeps the Score․ The theory posits that traumatic memories are not stored declaratively but in subcortical structures in the amygdala, hippocampus and brain stem, and trauma is remembered as impulses, autonomic dysregulations, and floods of affect rather than a narrative memory, bypassing the language center and Broca’s area․ The neurobiological perspective is extended by Laricchiuta et al. (2023), who state that the body keeps registering the effects of trauma long after the event that caused them.

Davidson et al. (2023) apply this trauma framework to oncology and argue that trauma-informed cancer care has clinical relevance․ In a scoping review of Scopus cancer literature, they have shown that cancer patients often experience post-traumatic symptomatology (intrusion, avoidance, and hyperarousal) in ways that mainstream cancer care often does not address. This clinical gap is what Boyer’s text dramatizes: the institution’s reduction of the patient’s complex traumatic experience to manageable data points. Consider Boyer’s chair of infusion as a moment of radical epistemological asymmetry: the repeated exhortations to “rate your pain on a scale of one to ten”, and her own conclusion that “sensation is the enemy of quantification.” (Boyer, 2019, p. 51).

One hermeneutical possibility, enabled by insights such as Van der Kolk’s, is to read the illness narrative’s syntactically fragmented, temporally disjunctive and affectively unsettled form as a literary figuration of implicit body memory: a way the form can gesture toward the non-verbal register of what trauma theory describes, without constituting clinical evidence of subcortical encoding as such (Laricchiuta et al., 2023)․ This hermeneutic possibility, novel to the emergent field of somatic literary criticism and informed by embodied cognition theory (Gallagher, 2005; Shusterman, 2008), holds that literary form can produce forms of embodied knowledge that analytical prose cannot relay, a possibility that Boyer’s text takes up self-consciously. This latter interpretive move is buttressed by advances in narrative neuroscience (Mar, 2011; Zunshine, 2006), which show that literary reading triggers embodied simulation and social cognition systems in the brain and may even afford us genuine understanding into somatic experience.

Another example of somatic inscription of trauma that looks to be a standard effect of chemotherapy, but is explicitly cited in medical literature, is “chemobrain.” Boyer provides the clinical images of patients taking the same treatment to show the “significantly reduced activation of the left middle dorsolateral prefrontal cortex and premotor cortex” with the treatment (Boyer, 2019, p․ 61)․ This citation shows Boyer’s autotheoretical methodology of combining biomedical research and phenomenological testimony with political critique (Carel, 2016; Toombs, 1992).

### 2.3 Biopolitics, precarity, grievability, and necropolitics

The politics of Boyer’s text are best framed within Judith Butler’s considerations of precarity and grievability in Precarious Life (2004) and Frames of War (2009)․ For Butler, precarity is the differential exposure to vulnerability or, in other words, the structurally uneven distribution of conditions of life and death along multiple axes of gender, ethnicity, class, sexuality, and nationality. Grievability affirms the normatively authorized range of lives that can be mourned, the deaths whose loss can be grieved. Grievability sets the terms for the deaths that are not mourned and therefore not grievable; it sets the conditions of invisibility of lives within the economies of public recognition and institutional response.

McCormack and Salmenniemi’s (2016) recent article in the European Journal of Cultural Studies on the biopolitics of precarity is a generative supplement to Butler’s analytics, as it foregrounds how neoliberal capitalist enterprises organize the logical institutionalization of precarity to deepen global and local inequities in health. The biopolitics framework elucidates Boyer’s depiction of her experience of cancer treatment, which likewise narrates the structural character of her situation: Cancer treatment in the account is organized “for the maximum profit of someone-not the patients” (Boyer, 2019, p. 65).

The concept of necropolitics, theorized by Mbembe (2003) in terms of the political production of death-worlds in which certain populations are made “living dead,” supplements her analytics by attending to the ethnicized components of biopolitical abandonment. Read together, Butler and Mbembe offer the tools for reading Boyer’s text in relation to the somatic experience of the narrator and the structural conditions of late-capitalist biomedicine’s differential economies of care and abandonment. Yet the application of “biopolitical care” to cancer by Ehlers and Krupar (2020) shows how institutional caring practices of oncology may support and discipline the cancer patient’s body, reproducing rather than contesting the structural inequities that constitute the experience of cancer. Boyer makes this contradiction clear in the simultaneous administration of life-prolonging and life-violating substances by institutional authority.

The pink-ribbon industrial complex of breast cancer culture, as Ehrenreich (2001) famously identified it, requires that cancer patients perform a specific affective script of gratitude, positivity, and transformation that renders them legible as grievable subjects within dominant cultural economies of recognition. Boyer refuses this script explicitly: “The nurses give me a glossy binder with a photo of a smiling silver-haired woman on its cover. The title is Your Oncology Journey, but I am certain that trip cannot be mine” (Boyer, 2019, p. 38). Her rejection makes Boyer illegible within the normative frameworks for public mourning of the cancer patient: visible as a living body and an object of research but invisible as a theorizing, resisting, politically indignant subject who refuses the demand for grateful compliance.

### 2.4 Intersectionality, global health equity, and feminist postcolonial theory

Thus, Boyer’s feminist illness narrative builds from the global stakes set forth by the WHO Global Breast Cancer Initiative (World Health Organization, 2023) and the United Nations Sustainable Development Goal 3 (United Nations, 2015)․ Each year, approximately 2․3 million people are diagnosed with breast cancer, and 670,000 die from it worldwide (World Health Organization, 2023)․ The most recent World Health Organization (2026) survival data reinforce how unequally disease burden is distributed․ Five-year survival estimates are an average of 87․3% when health systems are well-resourced, but are considerably lower at 41․9% in the lowest-income countries, with the lowest regional average being in the WHO African Region at 39․1%.

Estupiñán Fdez de Mesa et al. (2023) cite the intersection of gender, ethnicity, and class, and their collective role in cancer disparities that cannot be accounted for by clinical epidemiology in their scoping review protocol published in BMJ Open․ Similarly, Gutnik et al. (2022) refer to the differential breast cancer outcomes of African American and sub-Saharan African women as “a tale of global inequities”․ For instance, Unger-Saldaña (2014) traces similar disparities in low- and middle-income settings to “challenges to the early diagnosis and treatment of breast cancer”․ While the implications of ethnicity and geography for cancer care have been established as structuring factors in the literature on breast cancer biographies, Boyer’s memoir has little first-person account on the topic․ Close reading Boyer’s memoir will attend to gender and class, the structural axes on which the text provides the strongest textual evidence․ Ethnicity is a structural axis as well but is argued principally through the literature rather than through a close reading of Boyer’s memoir of the events.

At the same time, as González et al. (2023) argue, the act of storytelling can be articulated as a decolonizing act when it makes visible the structural conditions in which precarity is embedded. The question asked here, as part of Boyer’s autotheoretical affective labor, is: whose body becomes the assumed object of design in oncological treatment protocols, which are then scaled to a global level, and whose bodily experience is made invisible? Boyer’s write-up of this question is from the perspective of the world’s richest nation-state and a class of people typically subject to structural abandonment in poor-resource contexts․ Through this runs a summative economic argument: as Boyer argues, illness is situated in the ‘history of the world’, and embodiment amounts, as she writes, to ‘the history of what is done to most of us in the interest of the few’ (Boyer, 2019, p․ 24).

### 2.5 Phenomenology of illness and embodied subjectivity

Toombs (1992) phenomenological account of illness as a reordering of the lived body’s sense of time and space and Carel’s (2008, 2016) feminist phenomenology of illness are both meaningful for the epistemic status of Boyer’s first-person writing as a embodied oncological experience. Carel (2016) argues that illness constitutes a specific mode of lived-body knowledge unavailable to the healthy subject, which should not be reduced to anecdotal evidence, but instead viewed as epistemically authoritative. Boyer’s text thus does precisely this epistemological work: It makes a demand that the cancer patient’s somatic testimony be recognized as theoretical knowledge.

The phenomenological tradition of Merleau-Ponty (1962) and its application to illness by Toombs (1992) and Carel (2008) insist that the lived body is the original and fundamental mode of orientation to the world, and that to be ill is to have that orientation torn apart․ Boyer’s formal ingenuity is to transfer this syntactically ruptured mode of writing to the representation of this rupture in time․ She describes an exhaustion that dissolves appetite, desire, and finally sleep itself, “so fulminating that it is too exhausting for the body to relieve it” (Boyer, 2019, p․ 45).

### 2.6 Research gap

Though feminist illness narrative and autotheory studies are well established, *The Undying* has not inspired sustained academic engagement․ Critics have instead focused on Boyer’s formal opposition to survivor discourse and neoliberal discourses of positivity․ De La Parra Fernández (2021) reads the formal disruption as an attempt to reposition the cancer memoir against restitutive narrative conventions․ Fernández Morales’s (2020a, b) work on Ensler and Gubar offers a context for Boyer’s rejection of redemptive closure: a broader postfeminist critique of illness writing and the rhetoric of silence in autopathography․ This essay builds on this scholarship in two ways that have not yet been explored: by reading the formal fragmentation of the memoir in relation to trauma theory’s notion of implicit body memory rather than genre or rhetorical criticism and by considering the individualized witness of these fragments in relation to transnational regimes of health policy such as the WHO GBCI and UN SDG 3․ The neurobiological and somatic effects of trauma encoded in the technical form and effect of a text make for a different story. Whereas trauma studies has historicized ideas such as traumatic memory, belatedness, and representational rupture, literary studies has not yet considered in detail work from trauma neuroscience (e.g., Bessel van der Kolk’s study of subcortical body memory) in connection to the corporeal trauma of chemotherapy as an illness narrative. Yet, while Butler’s (2004, 2009) work on precarity and grievability has been productive for political and cultural theoretical work, the structural non-grievability of cancer patients within biomedical institutions has been less well explored within literary studies․ The cancer memoir is rarely part of global health equity initiatives (WHO Global Breast Cancer Initiative; United Nations Sustainable Development Goal 3)․ Framing *The Undying* as a somatic counter-archive of oncological trauma and institutional abandonment, this interdisciplinary essay inquires what the representation of oncological resistance can mean through trauma neuroscience, Butlerian grievability, feminist phenomenology, and global health.

## 3 Methodology

Combining insights from literary studies, medical humanities scholarship, feminist critical discourse analysis, and feminist policy analysis, this work presents a qualitative interdisciplinary model for reading *The Undying* (Boyer, 2019) as a text whose syntax, imagery, narrative frame, and affect together register somatic experience and constitute political argument․ It is important to stress that somatic testimony and political argument are the analytical lenses brought to the text in this study and are categories theorized out of trauma studies, feminist critical theory, and biopolitics, not any frameworks Boyer’s memoir sets out to impose on itself. This methodological distinction is important for what close reading, strengthened by these external frameworks, reveals about the text (and not necessarily what it reveals about Boyer’s own authorial intent).

Close reading proceeded by isolating (1) passages in which the narrator’s syntax or imagery registers a rupture in linguistic coherence at moments of somatic extremity (coded as evidence for traumatic body memory), (2) passages in which institutional actors dismiss, misname, or fail to register the narrator’s testimony (coded as evidence for precarity/non-grievability), and (3) passages in which material conditions of class and insurance status directly shape clinical treatment (coded as evidence for structural inequity).

The text of *The Undying* was read in full and coded for confirming, ambiguous, and counter instances․ Section 4 reports the analysis of the chemotherapy and treatment passages because they yielded the highest number of coded passages from any one category, not because the remaining passages were not coded at all․ Passages coded as ambiguous or counter instances were included using the same process as additions of confirming instances․ No counter instances were found in the dense segments of the memoir, and this limitation is discussed in Section 5․4 under the limitations of this corpus, and more specifically, restriction of the analysis to the treatment sections.

Literary close reading not only pays attention to what Boyer says but also how she says it: the fragmentation of syntax in moments of somatic extremity, the return to scenes of chemotherapeutic violence, and the refusal of narrative resolution. It employs a tradition of New Critical close reading that has been extended by feminist literary critics like Showalter (1985) and Gilbert and Gubar (1979) and phenomenologically oriented critics who have pioneered somatic literary criticism (Gallagher, 2005).

The reading of Boyer’s account that is undertaken here is one that is phenomenological in character, in the sense described by Toombs (1992) and Carel (2008, 2016). This is the sense in which what Boyer describes are descriptions of lived experience, offering an epistemological account of what it is to live in a body that is being institutionally violated. Phenomenological readings do not see Boyer’s account pathologically or as symptomatic but as an example of embodied knowledge that clinical epistemology systematically approaches but cannot capture (Davidson et al., 2023).

The study draws on feminist critical discourse analysis, Butler’s (2004, 2009) concept of precarity/grievability, and intersectional postcolonial feminist theories (Estupiñán Fdez de Mesa et al., 2023; Crenshaw, 1991; González et al., 2023) of ethnicity and ethnicization to describe how differential care, institutional neglect, and political economy of survivorship described by Boyer are articulated in terms of gender, class, and ethnicity․ The literary close reading is grounded in policy and regulation, including the WHO GBCI (World Health Organization, 2023) and the UN Sustainable Development Goal 3 (SDG) (United Nations, 2015).

### 3.1 Positionality statement

This study is not written from the lived experience of an oncological illness; instead, it reflects the perspectives of literary and medical humanities scholarship, feminist critical theory, and trauma studies. Therefore, it must be considered qualitatively different from Boyer’s own testimony, which is understood according to the critical techniques of those disciplines, including close reading and theoretical articulation, but this study lacks the first-person epistemic authority of her testimony. Consequently, this text does not supplant her testimony; rather, it can be read as an attempt at theoretical amplification․ The article attempts to preserve the specificity of the detail provided by her testimony, even as it is placed in dialogic tension with theoretical constructs.

## 4 Analysis

The analysis focuses on the chemotherapy, treatment, and somatic resistance sections, however, because they are Boyer’s most sustained and formally intense engagements with institutional violence: the sections of the book in which syntactic fragmentation, recursive return, and affective extremity manifest most strongly, and those that are therefore most legible and amenable to the close reading methodology with which it demonstrates their operations in Section 3․ Other aspects of the breast cancer apparatus, such as the relationship to the non-specialist clinic workers, the wellness and supportive care programming, and the patient navigation and survivorship/support group programs, less intensely covered in Boyer’s text, are outside the scope of this paper’s single-text close reading․ The Conclusion delineates various directions that future comparative work could take.

### 4.1 Traumatic body memory and chemotherapeutic inscription

As an interpretive heuristic, van der Kolk’s (2014) account of implicit, non-declarative body memory can act as a vocabulary through which to describe the work that the formal and esthetic elements of Boyer’s text perform (and thus, it should not be taken to be a claim about Boyer’s neurobiological status)․ *The Undying’s* broken prose, recursive time signature, and refusal of restitutive closure can be understood as a figuration of trauma’s resistance to articulation.

The longest sustained description of the somatic violence of chemotherapy in Boyer’s account is of Adriamycin (doxorubicin), a drug whose generic name she likens to the “brilliant and voluptuous red” of a ruby even as she notes its street names, “the red devil” and “the red death” (Boyer, 2019, p. 56). This conflation of poetic beauty and lethal danger enacts the more general logic of chemotherapy: an imperative to treat balanced by the need for caution with drug toxicity. The body cannot distinguish between a cure and a poison; both are perceived as an attack.

Building on this distinction, Boyer’s report of somatic experience is analyzed in three parts: formal/textual figuration, phenomenological report, and clinical explanation, which correspond (not very closely) to van der Kolk’s typology of implicit memory (subcortical encoding, physiological re-enactment, and affective residue)․ This typology is used here not as a theory concerning Boyer’s own neurobiology, but only as a descriptive analogy․ Boyer again speculates that the neurologic mechanism was sometimes dismissed as generalized cancer-related distress, a difficulty that oncology literature makes easier to dismiss since the mechanism is not clear: The blood–brain barrier excludes many chemotherapeutic agents, and it is unclear how a drug administered peripherally would act within the central nervous system (Ahles and Saykin, 2007)․ Boyer treats this as less a matter of settled mechanism than a lived reality (Boyer, 2019, p․ 61)․ For example, somatic testimony is institutionally silenced when the patient’s embodied knowledge is deemed epistemically irrelevant by biomedical authority, thus reproducing the structure of precarity that is, for Butler (2009), constitutive of non-grievable subjectivity.

However, this general pattern of epistemic injustice, in which clinician authority systematically discounts patients’ testimony and experience of their own bodies, is obvious throughout the epistemic injustice literature on a variety of conditions (Fricker, 2007, Carel and Kidd, 2014)․ The cultural apparatus of the breast cancer case is unique, however․ As Section 4․2 discusses, the kind of epistemic injustice built into the pink-ribbon survivorship script is not only the kind of clinical disbelief in one’s patient but also a positive affective demand (gratitude, positivity, and narratable recovery) that is institutionally and publicly visible in ways other forms of medical paternalism are not.

This is readily apparent in Boyer’s repeated visits to the IV room for chemotherapy infusion, “the cancer pavilion”, which she characterizes as a “cruel democracy of appearance” where all patients are reduced to a similar bald head and the same “plastic chemotherapy port visible as a lump under the skin” (Boyer, 2019, p․ 48)․ The leveling of difference that occurs in the context of clinical space is itself a form of institutional violence․ The person is reduced to a cluster of clinical signs, a data point in a medical database, or an object in a system rather than a subject inhabiting a world (Toombs, 1992; Carel, 2016)․ To take one further example, Boyer traces the origin of the second drug (cyclophosphamide) to “the chemical weapon Bayer developed under the name LOST” (mustard gas), which “lives on only in chemotherapy” (Boyer, 2019, p․ 68)․ This is not rhetorical․ It is the historical truth of the drugs injected into Boyer’s body that they contain the somatic memory of chemical warfare․ A subject receiving chemotherapy faces not only medically targeted violence but also sedimentary violence tied to a warlike past (Ehlers and Krupar, 2020; McCormack and Salmenniemi, 2016).

The amygdala, hippocampus, and prefrontal cortex show established short- and long-term changes after trauma․ These changes influence affect, cognition, and physiology later on (Laricchiuta et al., 2023)․ Clinically, such changes are not implausible․ Patients with chemotherapy-related cognitive impairment, Boyer writes, “lose the ability to read, to recall words, to speak fluently; they lose the ability to picture themselves in the past, to tell their story, to tell where they are, to know people they have known all their lives” (Boyer, 2019, pp. 62–63)․ Though such mechanisms are plausible from case studies, Boyer treats them not as evidence for a mechanism but as a metaphor.

### 4.2 Precarity, grievability, and the biopolitics of oncological care

Butler’s discussion of precarity and grievability also illustrates how Boyer’s particular conditions of treatment, not only her biography, can be an object of political analysis. Boyer’s working-class status as an uninsured patient is not incidental; it determines what care she receives, how she is treated in the clinical space, and whether her suffering is understood as sufficiently grievous to warrant public mourning and political redress.

According to Butler, being indexed to the axes of oncological access rendered by insurance, class, and ethnicity, these precarity types form a precarity structure․ This precarity structure enacts an uneven exposure to the risk of receiving ineffective or no treatment, being written off by institutions, and simply being abandoned by those institutions․ These terms in the following are used in the technical senses: precarity names the unevenly distributed structural exposure, epistemic injustice (Fricker, 2007; Carel and Kidd, 2014) names the actual mechanism of invalidating the patient’s testimony as a knowledge claim in the clinical encounter, and grievability names the downstream cultural and institutional effect of whether a given form of suffering counts as grievable and thus warrants public mourning and redress․ Thus, the dynamic in play here, as Boyer’s testimony shows, is that precarity determines who materially suffers from insufficient care, epistemic injustice determines whose account of that insufficient care is believed, and the combination of both is non-grievability, since a suffering that is both materially unaddressed and epistemically dismissed has no institutional mechanism for being recognized as what it is and mourned as such․ Boyer makes this precarity structure legible through a series of micro-narratives of clinical encounter. Boyer and her friends nicknamed her oncologist “Dr․ Baby, because of how much he resembled a cherub” (Boyer, 2019, p․ 25) who writes the steps of the diagnosis: hormone-positive, crossed out; HER2-positive, crossed out; triple-negative, confirmed․ The scene enacts the casualness with which institutional authority delivers devastating news to a patient with no buffer of class privilege to soften the blow.

The population-level evidence that breast cancer treatment varies by socioeconomic status and insurance coverage accords with Wheeler et al. (2013), who identify access to care, financial resources, and the quality of patient-provider interaction as potentially modifiable factors that influence whether a patient receives guideline-concordant care or not․ Silber et al. (2018) find that socioeconomic status predicts differences in breast cancer survival, even when the patient population is restricted to patients with Medicare and Medicaid․ Much of the disparity arises from differences in the presentation of breast cancer and differences in access to preventive care․ Boyer’s own condition as an uninsured working-class woman must thus be understood through this more widely documented structural condition․ At this point, it will be useful to delineate between experiences of Boyer that point to the structural conditions of under-resourced patients regardless of gender and those that take on a specifically gendered form․ Access to insurance coverage is obviously not gendered, but the work of translating bodies into clinical data, the cultural survivorship script, that is, in the argument that Boyer’s text advances, certainly is․ The latter are organized around and disproportionately bear upon women’s bodies and women’s illness narratives in ways that have no precise structural equivalent for male patients.

Consider the way that Boyer recounts the labor processes of the cancer pavilion․ with rare specificity, the gender of the oncological precarity is laid bare: women who “transmute bodies into data,” as the doctors interpret it, and Boyer herself, “a patient made of information, produced by the work of women” (Boyer, 2019, p․ 55)․ It is a feminist critique of the healthcare system’s reproduction of patriarchal hierarchies of knowledge and authority․ It is also a phenomenological acknowledgment of the reduction of the patient to data by care (Ehlers and Krupar, 2020; Manderson and Stirling, 2007).

Grievability thus illuminates what is arguably the most politically controversial aspect of Boyer’s book namely, her rejection, programmatically, of the cultural script of brave survivorship․ only those who survive, she notes, are “admitted to the pinkwashed landscape of awareness” in exchange for “5K runs, organic green smoothies, and positive thought” (Boyer, 2019, p․ 13)․ the critique of the pink-ribbon industrial complex identifies exactly the normative frame that Butler (2009) theorizes as grievability: a cultural imperative that cancer patients perform their suffering in ways that authorize, rather than challenge, the structural conditions that produce oncological precarity.

Like Ehrenreich (2001), two decades before, Boyer criticizes the materialist pink-ribbon critique of cheerfulness as conditional to the status of survivor; patients are expected to produce cheerfulness on demand․ she cites what this performance ignores: its “industrial etiology” and its “unequal distribution by class of the suffering and death of breast cancer” (Boyer, 2019, p․ 14).

### 4.3 Feminist micro-narratives of somatic resistance and autotheory

The third-mode Boyer’s text asks us to see is feminist micro-narratives of somatic resistance․ Boyer writes not of chemotherapy as a medical treatment and its side effects, but as an ontology, or way of being․ While describing the cancer pavilion’s patients, the narrator says: “the boundaries of our bodies break… there are nosebleeds and nausea and toxicity so extreme that we are told to flush the toilet twice․ we do not look like people: we look like people with cancer” (Boyer, 2019, p․ 48).

The section functions clinically, phenomenologically, and politically: as a report on and evocation of the body’s systemic reaction to such therapy and an account of how these are socially produced abject experiences. Drawing on the abjection of the cancer body (the excretion of toxic fluids and the disintegration of the physical boundary of the body, producing a visible, legible marker of its own disease), this may, following Kristeva (1982), be termed abjection: the dissolution of the self-other boundary and the subject-object distinction, which it is the task of the social order to contain. Boyer’s refusal to contain the abject, and her insistence on writing the cancer body’s dissolution is thus a political act: To narrate the cancer body’s dissolution is to make visible a form of bodily experience that the pink-ribbon industrial complex’s demand for a cheerful survivorship seeks to render invisible (Butler, 2009; Ehrenreich, 2001; Ehlers and Krupar, 2020).

Boyer’s autotheoretical method models a Medical Humanities method of considerable theoretical and practical importance. Evidence-based Medical Humanities research, such as Wang et al.’s (2023) demonstration that narrative medicine pedagogy can be shown to increase clinical empathy and patient-centered communication, has been disseminated widely. Boyer’s text models what might be called a more radically politicized form of this tradition, one that refuses the therapeutic domestication of illness narratives. Palla et al.’s (2024) scoping review of narrative medicine theory and practice in clinical and educational contexts confirms a long-standing tension in the field between approaches that have understood illness narrative as a resource for improving clinical work and those that understand it as a mode of political critique and structural analysis. Boyer’s text occupies the latter position with unusual force and theoretical sophistication.

As González et al. (2023) argue, storytelling can be a form of decolonizing practice when it makes visible the structural conditions that produce precarity. Boyer’s feminist micro-narratives of institutional abandonment accomplish this precisely. Boyer lists four failures: marginal patients at the margins of society, doctors’ failure to note continuing effects, coercion via financial hardship, and dismissing pain complaints according to gender․ Boyer also complains that routine billing practices publicize both the pain and the resources that support it․ The text’s refusal of the structure of beginning, crisis, and solution that typifies the restitution narrative enacts the political argument that ill health under late capitalism does not dissolve but remains a structural condition.

## 5 Discussion: medical humanities, biopolitics, and global health policy

### 5.1 Autotheory as medical humanities method

The study’s main sociological contribution (the central point developed in the preceding chapters) is the demonstration of three processes: that survivorship is an affective discipline of cancer patients, that women’s bodies and women’s institutional labor take part in the gendered organization of clinical labor that translates suffering into data, and that the translation of embodied suffering into standardized clinical data is a mechanism of institutional recognition and exclusion․ Boyer’s autotheoretical mode in the relation between embodied experience and critical theory is the literary strategy through which the study accesses and articulates this contribution, and it holds much promise for the field of Medical Humanities more broadly․ As Fournier (2021) has argued in relation to feminist literary practice, the autotheoretical genre rejects the partitioning of evidence and affect, data and body, and theory and experience that is so characteristic of both institutionalized practice and biomedical epistemology. For Boyer, autotheory exposes the political epistemology of illness: who qualifies as a knowing subject and a credible source of somatic testimony and whose suffering counts as legible and deserving of institutional support (see, e.g., Carel, 2016; Toombs, 1992).

As Palla et al. (2024) have noted in their scoping review, the classic tensions in narrative medicine are between those who see illness narrative chiefly as a tool for improving clinical practice and those who see it chiefly as a political critique and structural analysis․ Boyer’s text occupies the latter position with unusual force․ A Medical Humanities scholarship of this sort will arrange to hold together the phenomenological and the political, the individual and the structural, and the literary and the epidemiological․ A clinical analog to Boyer’s literary application of autotheory is Davidson et al. (2023) “trauma-informed” model of cancer care․ But both would agree that the cure for oncological trauma is not only a technical matter but also that of a structural critique of what has created the trauma.

### 5.2 Literary text and global health policy: a productive tension

While a mutually helpful relationship resides between the feminist micro-narratives of Boyer and the WHO GBCI (World Health Organization, 2023) and UN SDG 3 (United Nations, 2015) macro-level structures, it is not a relationship of complementarity, but rather one of what this study terms productive tension. It is in this way that the WHO epidemiological understanding of the global burden of breast cancer lays the statistical foundation for Boyer’s individual experience to attain transnational importance. What is not generated through the scientific method but is described elsewhere is the phenomenologically rich story of what it is like, in the first-person singular, to inhabit a body traversing the structural conditions that produce statistical inequalities (Estupiñán Fdez de Mesa et al., 2023; Gutnik et al., 2022; Unger-Saldaña, 2014).

As Estupiñán Fdez de Mesa et al. (2023) argue, intersectional analysis is necessary to understand cancer inequalities, including the multiple axes along which social determinants structure not only health outcomes but also the conditions of clinical encounters and institutional recognition. Boyer’s text constitutes a literary enactment of the kind of intersectional analysis that Estupiñán Fdez de Mesa et al. (2023) call for in the context of policy-oriented research. Boyer uses sustained somatic testimony and theoretical analysis to show how gender, class, and ethnicity are not merely social variables that affect cancer risk. They are also structural forces that condition care, produce institutional recognition and devaluation, and determine the cultural visibility of the cancer body’s suffering.

### 5.3 The postcolonial and transnational dimensions

Ultimately, reading a text by Boyer, a citizen of the richest country, as an American feminist illness narrative along with a transnational epidemiology might generate postcolonial questions. As an uninsured patient in the country of Economic superpower, Boyer holds a social class position that subjects her to a sort of institutional abandonment similar to what cancer patients in low- and middle-income countries face․ The comparison therefore is using structural similarity in the sense of similarity in degree, not kind, confined to structural similarity in the form of unequal access to treatment in a private healthcare system․ It is not capturing the very different histories of colonialism, healthcare systems, and access to global pharmaceutical supply chains that shape cancer treatment in the Global South and is therefore not methodologically supported by textual or empirical comparisons․ Keeping this caveat in mind, the World Health Organization (2023) data on survival by income setting can be placed in conversation with González et al. (2023) on decolonizing work in global oncology care: whose body is global oncology designed around and whose story is rendered invisible by the institutional processes through which global oncology claims to be addressing it?

By reading Boyer’s feminist micro-narratives of institutional abandonment through the WHO GBCI epidemiology data and González et al. (2023) new framework for decolonizing knowledge practices, the article argues that Medical Humanities, as a transdisciplinary field, is distinctly positioned to contribute to a postcolonial critique of global health governance. By showing how structural inequalities of gender, class, and ethnicity govern not only health outcomes but also the conditions of epistemic recognition—specifically, whose somatic testimony counts as evidence and whose suffering is legible as demanding redress —medical humanities can provide the catalysts for policy frameworks that meaningfully respond to the intersectional complexity of oncological experience.

### 5.4 Methodological limitations

Given that this analysis focuses on only one text, the concepts and practices put forward in this section must be understood as propositions generated from a close reading of one narrative, rather than generalizable data․ Single-text analysis also runs the risk of interpretive bias, since the interpretive frame applied to the text makes certain patterns visible and others invisible․ Close reading in this instance initially concentrated on those sections of the memoir in which this density of coded passages was largest, in this case with respect to those sections of the text concerning chemotherapy and treatment․ Although the full memoir was coded, counter-instances may be more frequent in sections not analysed in detail here․ Even given the limitations of extrapolating across memoirs, *The Undying’s* status as a Pulitzer Prize-winning, widely read cancer memoir gives it an unusual cultural reach and theoretical density that make it a productive site for this kind of sustained close reading․ Future work on the deployment of these strategies in other cancer memoirs, particularly those from the Global South, would shed further light on the claims advanced here.

## 6 Conclusion

This paper has read Anne Boyer’s memoir *The Undying* (2019) as a feminist archive, bringing oncological trauma, biopolitical precarity, and somatic resistance together․ Expanding upon the memoir, it has deployed Bessel van der Kolk’s (2014) neuroscientific framework of traumatic body memory and Butler’s (2004, 2009) analytics of precarity and grievability against the transnational epidemiological background specified by the WHO GBCI (World Health Organization, 2023) and UN SDG 3 (United Nations, 2015) to advance three interrelated arguments. First, it has argued that chemotherapy-related somatic violence is an institutionalized traumatic inscription to which the gendered body becomes bound and which it subsequently enacts beyond the clinical episode. Second, it has argued that Boyer’s cancer body is rendered structurally non-grievable by the normative epistemologies of late-capitalist biomedicine and breast cancer culture. Third, it has proposed that Boyer’s feminist micro-narratives of somatic resistance constitute a counter-archival practice that renders grievable what biopolitical regimes systematically exclude from the economies of institutional recognition and public mourning.

In Literary Studies, the paper shows that Boyer’s formal experimentation with syntactic fragmentation, recursive temporality, and the refusal of restitutive resolution can productively be read, through trauma neuroscience and feminist critical theory, as literary figurations of trauma’s resistance to verbal articulation rather than clinical enactments of a specific neurological process (Boyer, 2019; Laricchiuta et al., 2023; Caruth, 1996; Herman, 1992; Davidson et al., 2023)․ In Medical Humanities, the paper shows the productive convergence of literary close reading, phenomenological analysis, feminist critical theory, and policy contextualization and their capacity, together, to hold together the individual and the structural, the somatic and the political (Palla et al., 2024; Wang et al., 2023; Charon, 2001). In the context of Global Health Policy, without humanistic frameworks that render visible the structural mechanisms by which gender, class, and ethnicity determine not only health outcomes but also the conditions of clinical encounter, institutional recognition, and epistemic authority, the WHO GBCI’s (World Health Organization, 2023) equity imperatives and UN SDG 3’s mandate of gender-equitable oncological care cannot be fully realized (Estupiñán Fdez de Mesa et al., 2023; Gutnik et al., 2022; González et al., 2023).

Future work might, for example, compare Boyer’s text to contemporary cancer memoirs in feminist literary traditions in the Global South, generating a stronger transnational and postcolonial account of oncological grievability. The neuroscientific model adapted from van der Kolk’s, 2014 work on subcortical traumatic encoding might also be fruitfully applied to the formal deployment of illness narrative more broadly, offering a basis for reading the relationship between literary form and somatic testimony in other contexts. Finally, the productive tension between literary-humanistic analysis and global health policy that this paper has identified suggests the need for formal, interdisciplinary infrastructures in which Medical Humanities scholars, epidemiologists, clinicians, and global health policymakers can work together to develop integrative approaches to equity imperatives like those enshrined by SDG 3 and the WHO GBCI.

## Data Availability

The original contributions presented in the study are included in the article/supplementary material, further inquiries can be directed to the corresponding author.
